# Rare presentation and retrograde diagnosis of total colonic aganglionosis in a female infant: a case report

**DOI:** 10.1186/s13256-023-03832-1

**Published:** 2023-04-08

**Authors:** Konstantine Chakhunashvili, Davit G. Chakhunashvili, Eka Kvirkvelia, Eka Gozalishvili

**Affiliations:** 1 The University of Georgia, Kostava 77a, Tbilisi, 0171 Georgia; 2 Evex Hospitals – Irakli Tsitsishvili Children’s Clinic, Lubliana 23, Tbilisi, 0179 Georgia

**Keywords:** Total colonic aganglionosis, Enterocolitis, Hirschsprung disease

## Abstract

**Background:**

Total colonic aganglionosis is an extremely rare variant of Hirschsprung’s disease, which is predominant in males and can be seen in 1:50,000 live births. The presented case not only depicts a rare case, but also unusual clinical, laboratory, and instrumental data.

**Case presentation:**

A 2-day-old Caucasian female newborn was transferred to our hospital from maternity. The initial presentation was reverse peristalsis, abdominal distention, and inability to pass stool. Fever had started before the patient was transferred. Hirschsprung’s disease was suspected, and tests such as contrast enema and rectal suction biopsy were done. Before enterostomy, the management of the disease included fluid resuscitation, colonic irrigation, antibiotic administration, enteral feeding, and supportive therapy. During ileostomy operation, no transition zone was visualized and full-thickness biopsy samples were retrieved from the rectum and descending colon. After surgical intervention, status significantly improved—defervescence and weight gain most importantly improved.

**Conclusion:**

It is well known that diagnosis of total colonic aganglionosis may be delayed for months or even years since the transition zone may not be visible and rectal suction biopsy, unlike full-thickness biopsy, is not always reliable. It might be more prudent not to be derailed because of negative radiography and rectal suction biopsy. Also, doctors should be more suspicious of the disease if signs and symptoms are starting to be consistent with Hirschsprung-associated enterocolitis, despite biopsy and radiology results.

## Introduction

Hirschsprung’s disease (HD), also referred to as congenital aganglionic megacolon, is associated with the complete absence of ganglion cells from colonic nerve plexuses (Auerbach and Meissner). Aganglionic regions of the colon cannot relax properly and become spastic over time, which ultimately leads to intestinal obstruction of the distal colon [1]. Regarding the length of the aganglionic region, Hirschsprung’s disease can be classified into three groups: total colonic aganglionosis (TCA), short-segment aganglionosis, and long-segment aganglionosis [2]. TCA is very rare and accounts for about 5% of all HD cases (predominantly men) and can be seen in 1:50,000 live births [3]. HD can clinically manifest within a few days after birth, with meconium ileus, bilious vomiting, and distended abdomen [4], while encopresis, hypoproteinemia, enterocolitis, and empty rectum on digital examination in conjunction with poor weight gain can also occur [3]. Diagnosis of HD should be excluded in every infant and child with constipation [5].

## Patient information

A 2-day-old Caucasian female patient has been transferred straight from maternity to our hospital. The main concern was reverse peristalsis, together with significant abdominal distention and inability of passing stools with fever. On the same day, an adequate stimulation resulted in defecation, reverse peristalsis stopped, and abdominal distention (circumference) was reduced. Enteral feeding resumed; however, the patient could not pass stools without stimulation. Within 2 days of the first contrast enema, which was performed on day 4, the patient experienced the start of a high fever.

There was no family history of Hirschsprung’s disease mentioned, nor other genetic conditions present suggesting it. Before initiating contrast enema, a rectal examination was done, and no explosive gas and/or fluid release was detected; no feces were in the rectum either. Later on, the status started to deteriorate, and fever became more aggressive, heart and respiratory rate increased, and livedo reticularis started to predominate together with significant abdominal distention, which as time passed, did not reduce with defecation. A digital rectal examination became positive for the explosive release of gas after 3 weeks of hospitalization at our hospital.

## Timeline and diagnostic assessment

Delay in meconium passage, abdominal distention, and reverse peristalsis prompted medical team to suspect Hirschsprung’s disease and perform a contrast enema (on day 4), and rectal suction biopsy (on day 10). Even though no abnormalities were found by either contrast enema or rectal suction biopsy, we could not assign a diagnosis of functional constipation, since without colonic irrigation no stools were being passed, and we had evidence of protein wasting and failure to thrive, despite the adequate feeds. Meconium plug syndrome was excluded, since contrast enema is also a therapeutic measure in this condition. However, in our case the symptoms persisted, regardless [6]. The diseases that might be a cause for functional constipation were either not suspected because of the course of the disease (lead poisoning, chronic intestinal pseudo-obstruction, and so on) or excluded (hypothyroidism, spinal dysraphism, intestinal neuronal dysplasia, and so on) [7, 8].

After contrast enema, fever became high and obvious signs of systemic inflammatory response syndrome were present. Though these events came after the contrast enema, and a connection should have been made to Hirschsprung-associated enterocolitis right away, negative histology and radiology results blinded us to this diagnosis, which was a major mistake. On days 35–40 of hospitalization, neonatal necrotizing enterocolitis in term infants, an exceptionally rare diagnosis, was also being considered, however, radiographic and clinical evidence were against it, since only abdominal distention was present and deterioration in case of this disease is much faster than observed in the case of our patient [9]. Eventually, mounting evidence, such as the radiographic evidence —Kloiber cups, extreme enterocolonic flatulence, and megacolon, the clinical evidence—abdominal distention not subsiding with defecation, digital rectal examination becoming explosive for gas, high fever, and failure to thrive, and the laboratory evidence—protein wasting, and increased inflammatory markers, all pointed to Hirschsprung-associated enterocolitis (grade II). Surgical intervention, namely an enterostomy, was done 75 days after hospitalization [10].

### Radiography

**Fig. 1:**
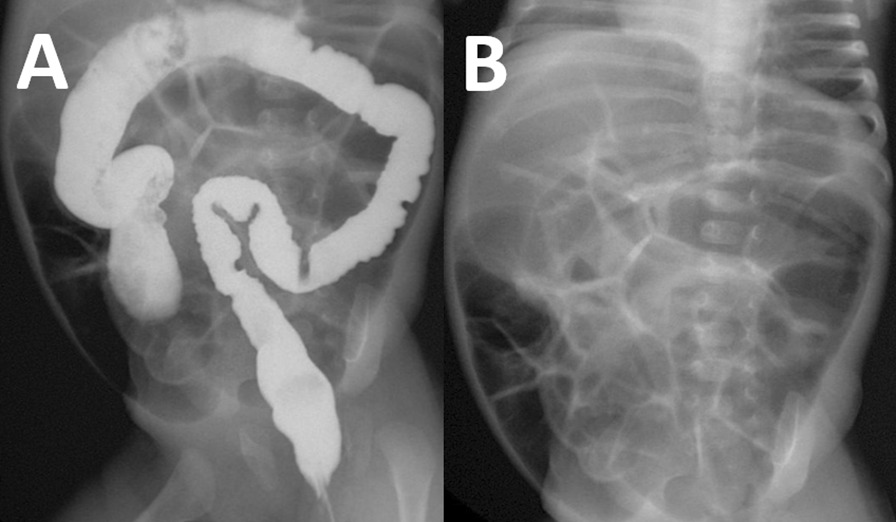
**A** Transition point cannot be visualized via barium enema (day 4 after hospitalization). **B** Anteroposterior radiograph demonstrating significant enterocolonic flatulence (day 4 after hospitalization)

**Fig. 2:**
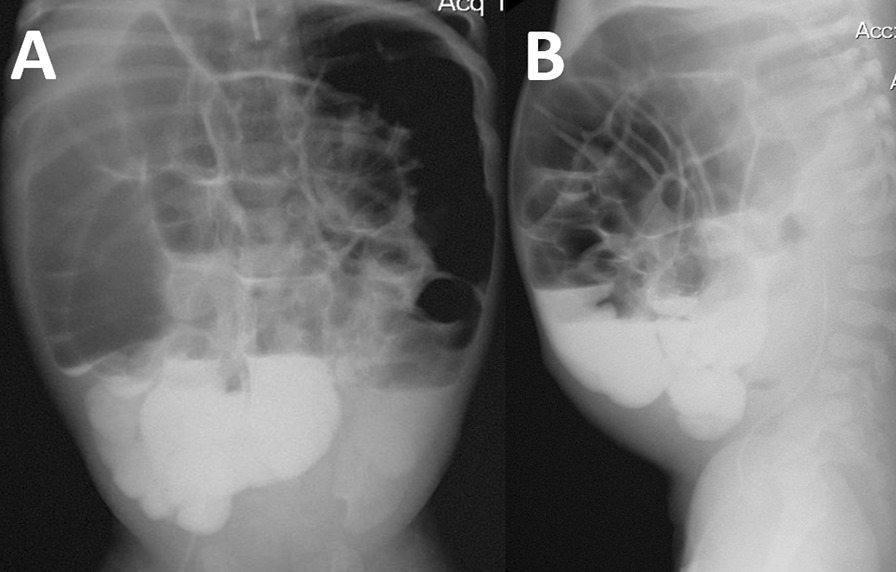
Anteroposterior (**A**) and lateral (**B**) oral contrast radiographs depicting Kloiber cups, flatulence, and megacolon (day 75 after hospitalization)

Contrast enema was done on day 4 of hospitalization, and did not show a transition zone (Fig. 1). About a month later, plain anteroposterior radiography showed extensive colonic and intestinal flatulence (Fig. 1). Before, intervention per oral contrast radiography demonstrated extreme meteorism, Kloiber cups, and signs of megacolon (Fig. 2).

### Laboratory assessment

**Fig. 3:**
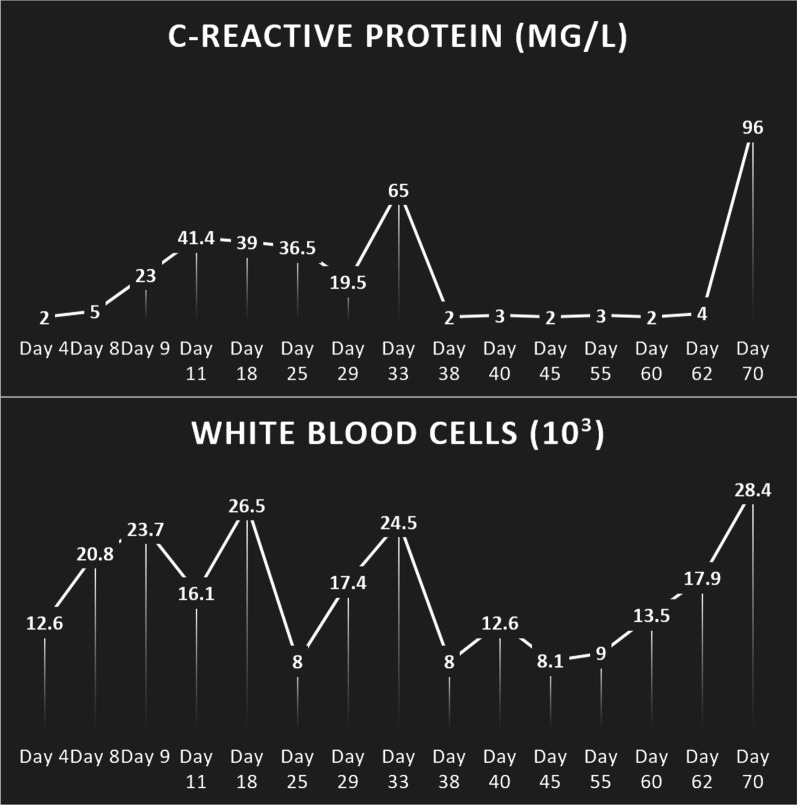
This figure depicts levels of C-reactive protein (CRP) and white blood cells (WBC) from day 4 to day 70 after the patient was hospitalized, right before surgical intervention. We can see significant increase in WBC and CRP levels after contrast enema for about a month, and then stable decline of CRP and WBC up until day 70 when levels suddenly surged

Laboratory assessment showed extremely high inflammatory markers; however, cultures of the blood, urine, and feces were negative every time (Fig. 3). Protein wasting was documented, which was corrected with intravenous infusions.

### Biopsy

**Fig. 4:**
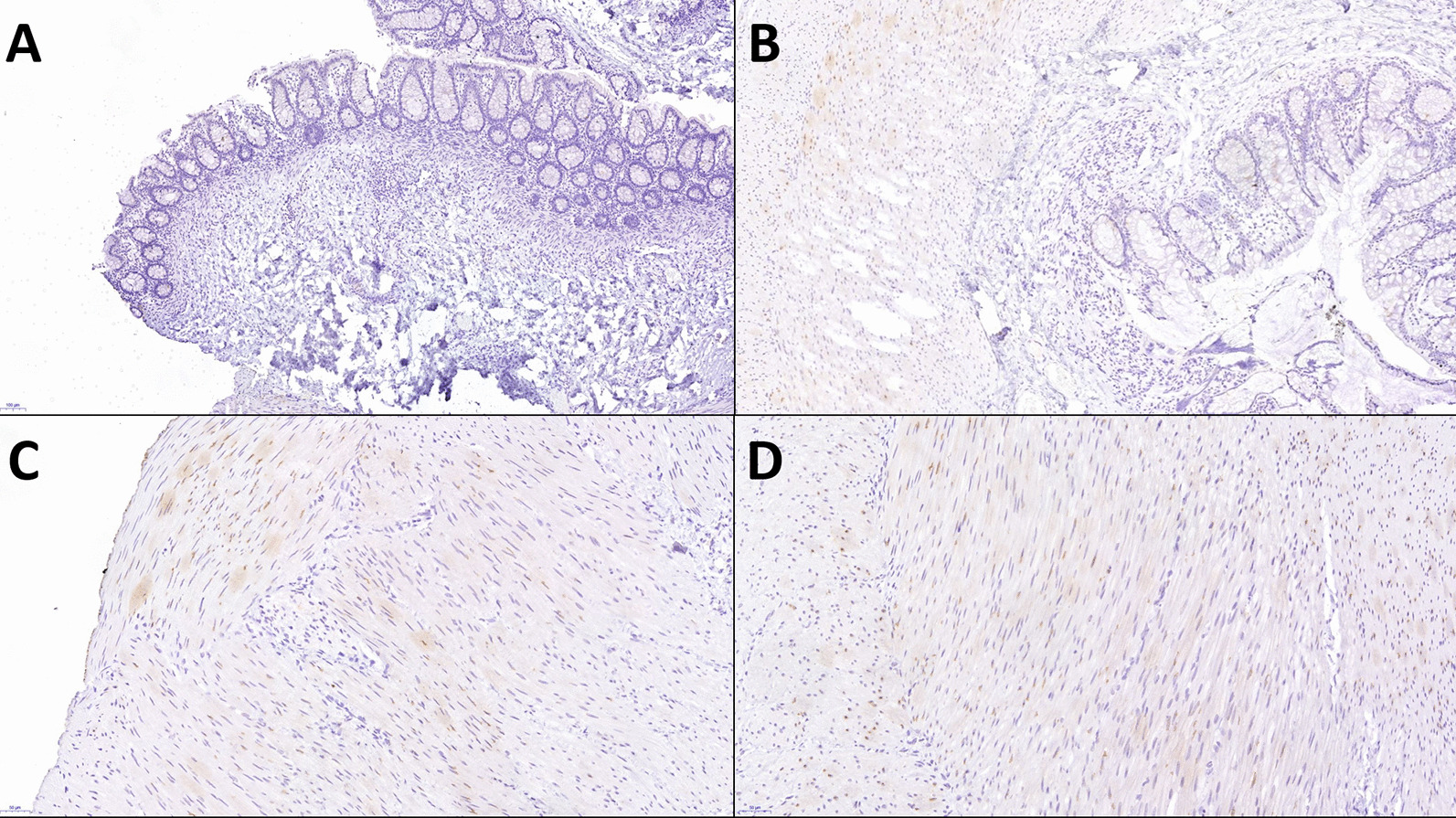
In this figure we can see magnified rectal biopsies stained for calretinin. The pictures are magnified at ×8.9, ×9.6, ×21, ×22.6 for **A**, **B**, **C**, and **D**, respectively. We can see absence of ganglion staining, meaning that calretinin-immunoreactive mucosal innervation is absent

**Fig. 5:**
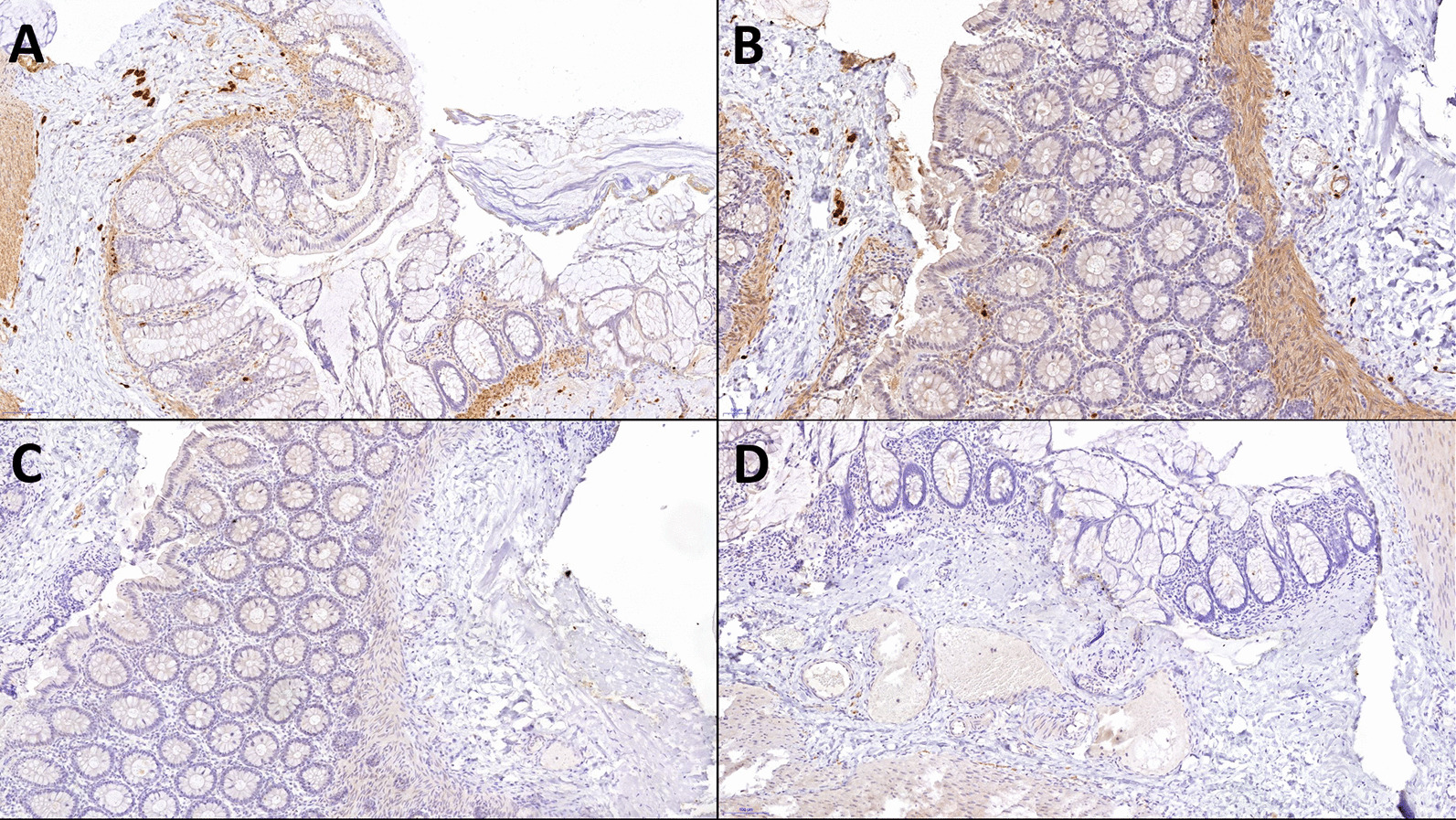
**A**, **B** In these pictures, which depict a biopsy specimen stained for S-100, neural hypertrophy and increased muscular mucosa tissue are demonstrated (magnification ×15.1 for **A**, and ×18.3 for **B**). **C**, **D** Staining with neuron specific enolase (NSE) clearly demonstrates an aganglionic picture (magnification ×11.2 for **C**, and ×15.9 for **D**)

The first rectal biopsy was performed on the day 10. However, pathologists could not confirm the diagnosis. The second biopsy specimens, full-thickness samples from the rectum and colon, were subjected to histology and immunohistology staining. As a result, total colonic aganglionosis was confirmed via pathologic examination (Figs. 4, 5). The specimens for the second biopsy were retrieved during surgical intervention.

## Therapeutic intervention

Before enterostomy, the disease management included fluid resuscitation, colonic irrigation, antibiotic administration, enteral feeding, and supportive therapy. While ileostomy was being performed, no transition zone was visualized. After surgical intervention, within 14 days, fever completely abated, and over 4 weeks significant weight gain (2 kg) was observed, both of which were a clear indications of clinical improvement.

## Follow-up

So far, no enterostomy-related complications have been observed. The infant is passing routine visits for healthy child weight gain and catch-up growth monitoring. The decision about closure enterostomy and colectomy will be made at 12 months of age.

## Discussion

Different diagnostic approaches such as barium enema, anorectal manometry, and biopsy can all aid in diagnosis, although anorectal biopsy remains the gold standard [1]. Anorectal manometry can be hard to perform in infants [3]. The transition zone, which is the hallmark of HD, can be detected with the help of barium enema, and is hard to visualize in most cases [1]. As the normal bowel segment needs some time for dilation, the probability of diagnosing HD with contrast enema is higher in children above the age of 1 month [4]. A significant amount of retained contrast in the intestines after 24-h delayed films can also increase suspicion about HD [4]. Histopathological diagnosis of HD can be made by confirming the lack of ganglion cells in the submucosal plexus in the samples taken with the help of a biopsy from variable regions of the colon [11, 12]. Although biopsy continues to be a gold standard, diagnosis of HD remains a challenge, especially in neonates, as ganglionic cells of submucosa are hard to identify due to lack of differentiation and small size [11]. Characteristic features of cytoplasm and neuronal nucleus may also be missed [12]. Without a timely diagnosis, acute enterocolitis or toxic megacolon can occur before or after surgical repair [1, 13]. Resection of the aganglionic segment of the colon, followed by temporary ostomy is the main treatment option for HD [3]. In this case report, we describe a highly rare variant of HD with highly rare clinical, laboratory, and instrumental results.

Upon admission of this patient, there was a high clinical suspicion of HD due to the presence of abdominal distention, reverse peristalsis, and inability to pass stools. At first, there was no firm objective evidence for Hirschsprung-associated enterocolitis (HAEC) [14]. Even though there was no explosive gas or fluid release, and plain radiography showed no evidence of HAEC at first, the fever had already started. The contrast enema and punch biopsy were performed to confirm the diagnosis of HD [15]. The radiographic imaging after the enema did not show a transition point, and the histology results could not yet confirm the diagnosis.

Even though biopsy is a gold standard for making a final diagnosis of HD, it is well known that in infants it is quite challenging to confirm the diagnosis through rectal suction biopsy, as opposed to full-thickness biopsy [16].

Subsequently, clinical and laboratory evidence showed symptoms and signs of systemic inflammatory response syndrome (SIRS), with the condition stabilizing upon antibiotic use. However, as soon as it was discontinued the symptoms and signs promptly resumed. Since the patient did have a high fever prior to the contrast enema, we can deduce that contrast enema induced bacterial translocation and sepsis. This is quite frequent in HAEC, and why it is prudent to avoid contrast enema if the latter is suspected [3, 17–19].

Other presenting features of HD were hypoproteinemia and failure to thrive, which were both evident, hence, protein transfusion was done in the ICU. However, weight gain was practically absent [3].

There are other diseases that should be considered, such as celiac disease, meconium plug syndrome, chronic intestinal pseudoobstruction, gastrointestinal malformations, malrotation, etc. Although, some of them can be diagnosed or treated by radiologic studies and have different clinical features, the absence of ganglia is the strongest argument for HD [10, 20–22].

The main reason for the surgical delay was absence of histological evidence. However, over time, evidence became overwhelmingly in favor of HAEC: extensive enteral and colonic flatulence, a rectal digital examination positive for explosive gas release, unremitting fever, and severely dilated descending colon with a surge of inflammatory markers; the last two might have hinted at the start of toxic megacolon, which prompted the medical team to move forward with enterostomy.

After the surgery, after about 2 weeks, the clinical and laboratory signs of improvement were evident, such as weight gain, defervescence, and passing stools through the stoma without assistance.

It has been well known that TCA is hard to diagnose in infants and diagnosis can be delayed for months or even years. However, this case suggests that sometimes it might be wise to go forward with interventions sooner and not be derailed because of inability to visualize the transition zone or negative rectal suction biopsy results [23, 24]. Since there were mountains of evidence that the transition zone may not be visible, especially in TCA and rectal suction biopsy, as opposed to ful-thickness biopsy, it is not a reliable tool for exclusion [25, 26]. Sarin *et al.* also reported the similar concern in 2014, but they mostly focused on the perils of resource-poor countries encountering and managing TCA [27].

## Conclusion

First of all, if fever is evident and HD is suspected, it would be wiser to rigorously assess the risk–benefits of contrast enema, since it can cause bacterial translocation and sepsis. Secondly, the medical team should not be thrown off course because of negative results on the rectal suction biopsy and the absence of a transition zone. It should be made clearer for everyone that this type of investigation is good for confirmation, but cannot be used to rule out the disease. Thirdly, physicians should always have in mind that HAEC, rarely, can be one of the first signs of HD. In the end, we do think that systematic review and implementation of the standardized diagnostic approach regarding HD, which will encompass all the rare cases, will aid doctors worldwide.

## Data Availability

[REAGENTS/TOOLS/MATERIALS] generated in this case report are available from the corresponding author upon request.
